# Recurrent Pyelonephritis in Quadriplegia: Rethinking Oral Cefdinir in the Presence of Nephrolithiasis

**DOI:** 10.7759/cureus.102477

**Published:** 2026-01-28

**Authors:** Mahsum Jafri, Zuhayr Khan, Constantino G Lambroussis

**Affiliations:** 1 Internal Medicine, Lake Erie College of Osteopathic Medicine, Elmira, USA; 2 General Medicine, Lake Erie College of Osteopathic Medicine, Elmira, USA; 3 Osteopathic Medicine and Family Medicine, Lake Erie College of Osteopathic Medicine, Elmira, USA

**Keywords:** acute pyelonephritis, cerebral palsy, recurrent nephrolithiasis, recurrent uti, renal calculi

## Abstract

Patients with cerebral palsy who develop functional quadriplegia are at an increased risk for recurrent urinary tract infections and kidney stones. Impaired bladder emptying, chronic constipation, and reduced physical mobility collectively promote urinary stasis and bacterial growth. Despite the frequency of these complications, there is little guidance in the currently available literature on how to select appropriate empiric oral antibiotics for pyelonephritis in this patient population, especially when renal stones are also present. This case highlights the importance of careful antibiotic selection in patients with neurogenic bladder and nephrolithiasis. Oral cefdinir demonstrates limited renal tissue penetration, which may reduce its effectiveness in treating complicated urinary tract infections. In contrast, fluoroquinolones provide superior penetration into renal tissue, making them a more suitable option in specific scenarios. Choosing antibiotics that align with the patient's clinical presentation and pharmacological requirements can significantly reduce recurrence and improve outcomes in quadriplegic patients with recurrent kidney stones. This case report demonstrates that selecting antibiotics with sufficient renal tissue penetration can improve outcomes in complicated pyelonephritis, with symptom resolution after antibiotic change.

## Introduction

Patients with quadriplegia often face multiple overlapping medical challenges, including neurogenic bladder and bowel dysfunction. These factors are well recognized to predispose quadriplegia patients to urinary stasis, chronic constipation, and recurrent nephrolithiasis. These interrelated conditions create a cycle of urinary retention, infection, and inflammation that remains poorly addressed within the current available literature. Chronic constipation increases intravesical pressure by compressing the bladder, which can result in elevated post-void residual volumes and promote bacterial ascent within the urinary tract [1]. In addition, gut dysbiosis may alter urinary oxalate metabolism and calcium absorption, thereby increasing susceptibility to recurrent kidney stone formation [2]. Once renal calculi develop, they frequently serve as bacterial reservoirs that harbor urease-producing organisms, such as Proteus and Klebsiella [3]. These bacteria thrive in alkaline urine and contribute to the formation of struvite stones. They also generate biofilms that limit antibiotic diffusion, resulting in persistent or relapsing episodes of pyelonephritis [3]. In such scenarios, antibiotic pharmacokinetics and tissue penetration become critical determinants of treatment success. Despite the frequency of these overlapping conditions, there remains limited guidance on empiric oral antibiotic selection for pyelonephritis in quadriplegic patients with recurrent nephrolithiasis.

Oral third-generation cephalosporins, such as cefdinir, are often prescribed for outpatient management. However, their limited bioavailability and poor urinary penetration may reduce therapeutic efficacy of third-generation cephalosporins. Recent work by Mitzner et al. (2025) reported that patients treated with cefdinir for uncomplicated urinary tract infections had nearly double the failure rate than those receiving cephalexin, raising concerns about the effectiveness of first-line cephalosporins in clinical practice [4]. In contrast, fluoroquinolones demonstrate excellent oral bioavailability of 99%, high renal excretion of approximately 87%, and proven activity against common Gram-negative uropathogens [5]. Despite these differences, there are no clear clinical guidelines to direct empiric oral antibiotic selection for patients with quadriplegia with concurrent nephrolithiasis. According to a 2016 article by Vigil and Hickling, neurogenic bladder patients, including those with quadriplegia, require individualized, culture-guided antibiotic therapy along with urodynamic studies (UDS)/video urodynamic studies (VUDS), ultrasound (US), and cystoscopy to evaluate for modifiable factors, such as stones and altered bladder physiology [6]. The following case highlights the clinical implications of antibiotic choice in this unique high-risk population.

## Case presentation

A 26-year-old woman with functional quadriplegia secondary to cerebral palsy was brought to the emergency department by her family. She had ongoing right-sided abdominal pain for three days and poor oral intake. Due to her neurological limitations, the patient was nonverbal, and her family provided the history. The family noted that she had appeared increasingly uncomfortable, had eaten very little, and intermittently guarded her abdomen. There were no reports of fever, vomiting, or chills. In addition to cerebral palsy with quadriplegia, the patient’s medical history additionally consists of seizure disorder, severe chronic constipation, and recurrent nephrolithiasis for which she has previously undergone two ureteral stent placements. There were no additional significant medical comorbidities. Her chronic medications were reviewed and were not felt to contribute to her infectious presentation or clinical course.

On initial presentation to the emergency department, she was noted to be tachycardic but otherwise hemodynamically stable. Physical examination revealed tenderness in the right lower quadrant radiating toward the flank. Laboratory studies demonstrated hypokalemia at 2.9 mEq/L, hypomagnesemia at 1.6 mg/dL, with a non-elevated white blood cell count. Urinalysis was positive for nitrites, leukocyte esterase, and 3+ white blood cells.

**Figure 1:**
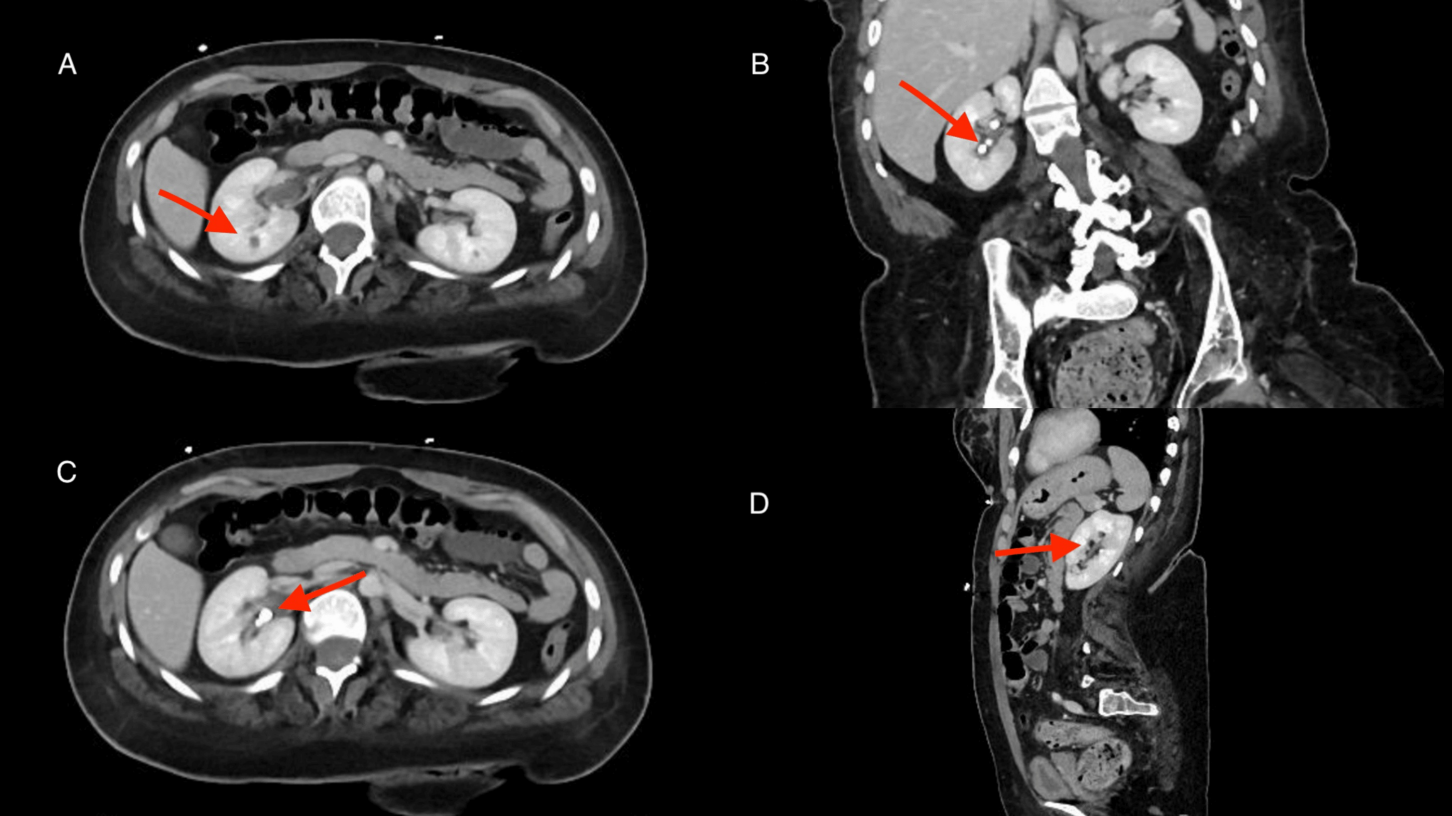
Contrast-enhanced CT abdomen and pelvis demonstrating acute pyelonephritis and right nephrolithiasis. (A) Axial contrast-enhanced CT with right perirenal fat stranding and a hypoenhancing cortical focus in the anterior mid-right kidney, consistent with pyelonephritis. (B) Coronal reconstruction showing multiple right renal calculi. (C) Axial CT with non-obstructive right renal stones, pyelonephritis without obstruction. (D) Sagittal reconstruction with longitudinal view of the right kidney with a visible region of focal hypoenhancement consistent with pyelonephritis.

A contrast-enhanced CT scan of the abdomen and pelvis showed right perirenal and periureteral fat stranding along with a small hypoenhancing focus in the anterior mid-right kidney, consistent with acute pyelonephritis (see Figures 1A, 1D). Multiple right renal calculi were present on coronal and axial reconstructions, with the largest measuring 0.7 cm (see Figures 1B, 1C), along with a 1.1 cm right lower-pole lesion of higher attenuation than simple fluid, likely representing a complex cyst (Figure 1A). No hydronephrosis or ureteral obstruction was identified.

The patient received 2 l of intravenous normal saline, 1 g of intravenous ceftriaxone, acetaminophen, and potassium chloride 40 mEq. She was admitted and started on intravenous cefepime for broader Gram-negative coverage. Blood cultures remained negative. A urine culture obtained on the initial visit grew a Gram-negative organism, with final speciation and antimicrobial susceptibilities finalized two days later. During her hospitalization, she experienced intermittent low-grade fevers that resolved with acetaminophen and was given phenazopyridine for urinary discomfort. Electrolyte abnormalities were corrected. With clinical improvement, our patient was discharged home. She was prescribed oral cefdinir 300 mg twice daily for seven days as empiric outpatient therapy while final culture susceptibilities were pending. She was also prescribed oxycodone-acetaminophen 5-325 mg tablets every six hours as needed for pain relief, and phenazopyridine 100 mg tablets for three days.

Eight days after discharge, she returned with persistent flank pain and new-onset transaminitis. Laboratory studies showed mild transaminitis with aspartate aminotransferase (AST) 83 U/L (reference range: 8-48 U/L) and alanine aminotransferase (ALT) 174 U/L (reference range: 7-55 U/L). Our patient remained afebrile during this encounter. Urinalysis was again positive for nitrites, positive for leukocyte esterase, and urine culture on readmission grew *Escherichia coli*. 

**Figure 2:**
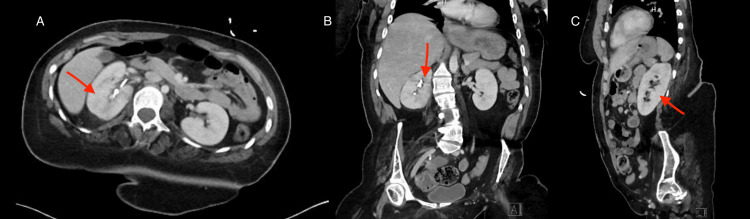
Follow-up contrast-enhanced CT demonstrating interval improvement of right-sided pyelonephritis. (A) Axial contrast-enhanced CT improving cortical enhancement with mild perirenal fat stranding. (B) Coronal reconstruction of multiple renal calculi in the right pelvis, resolving pyelonephritis. (C) Non-obstructive right nephrolithiasis and improving cortical inflammatory changes consistent with pyelonephritis.

Urology was consulted, and she underwent cystoscopy with right ureteral stent placement for recurrent nephrolithiasis and pyelonephritis. Following the procedure, she was transitioned to levofloxacin 500 mg daily for seven days, along with phenazopyridine and tamsulosin. A follow-up contrast-enhanced CT scan showed improvement in the right-sided pyelonephritis with improvement in cortical enhancement and decreased perirenal fat (see Figure 2A). Coronal reconstructions showed persistent renal calculi and improving inflammatory changes (see Figures 2B, 2C). Her liver enzymes, which were initially elevated AST 83 U/L (reference range: 8-48 U/L) and ALT 174 U/L (reference range: 7-55 U/L), normalized on repeat testing to AST 18 U/L (reference range: 8-48 U/L) and ALT 38 U/L (reference range: 7-55 U/L), her pain resolved, and she was discharged home in stable condition.

## Discussion

Patients with functional quadriplegia secondary to cerebral palsy often experience overlapping urologic and gastrointestinal complications, including neurogenic bladder, impaired bowel motility, and recurrent nephrolithiasis. These conditions interact to promote urinary stasis and infection risk, creating a complex setting where antibiotic choice can greatly affect outcomes. Chronic constipation and neurogenic bowel dysfunction increase bladder pressure through rectovesical compression, which raises post-void residuals and allows bacteria to ascend the urinary tract [1]. Gut dysbiosis in patients with poor bowel motility can disrupt urinary oxalate metabolism and calcium absorption, predisposing them to recurrent kidney stones [2]. Once calculi form, they can become reservoirs for bacteria, such as Proteus and Klebsiella, organisms capable of producing urease and forming biofilms. Biofilms limit antibiotic diffusion and contribute to recurrent or relapsing pyelonephritis [3]. In such situations, understanding the pharmacokinetic and pharmacodynamic properties of the chosen antibiotic becomes crucial for treatment success. 

Patients with spinal cord injury or quadriplegia often face complex interactions between urinary tract physiology and infection risk. Impaired bladder emptying, chronic catheterization, and altered immune responses create a clinical environment where bacteria can easily persist or recolonize. Treatment practices for urinary tract infections in individuals with spinal cord injury vary widely across institutions, reflecting the lack of strong evidence and standardized recommendations. Empiric antibiotic therapy in this population is further complicated by atypical presentations, such as the absence of fever or dysuria, and by frequent colonization with multidrug-resistant organisms [3].

Compounding these challenges, urolithiasis is significantly more common in patients with spinal cord injury due to recurrent infections, immobilization, and long-term catheter use. Stones not only act as bacterial reservoirs but also perpetuate a cycle of infection and inflammation that can compromise renal function over time [3]. Patients with complete spinal cord injuries, such as those with quadriplegia, who have profound immobility, have an increased risk for nephrolithiasis. Prolonged immobility accelerates bone resorption, leading to hypercalciuria, resulting in stone formation. Limited mobility is another factor contributing to urinary retention, fostering an environment for calculi development. Additionally, more than 60% of quadriplegic patients require catheterization, increasing the rate of urinary tract infections and stone formation [7]. This interplay between neurogenic bladder, urinary stasis, and calculi formation underscores why antibiotic therapy must target both free-floating and biofilm-embedded pathogens. The presence of stones alters the pharmacologic landscape of infection, as certain agents penetrate poorly into the surrounding renal tissue [3]. Therefore, antibiotic choice in this subset of patients requires balancing microbial coverage with tissue pharmacokinetics.

Cefdinir, a commonly used oral third-generation cephalosporin, has a relatively low oral bioavailability of approximately 21% [8]. Although cefdinir provides broad Gram-negative coverage, these pharmacologic limitations can reduce its effectiveness in complicated urinary infections and pyelonephritis, especially when stones are present. By comparison, fluoroquinolones, such as levofloxacin, achieve near-complete oral absorption greater than 99%, and high renal excretion of approximately 87%, with proven activity against typical uropathogens [5]. Fluoroquinolone's superior tissue and biofilm penetration make them better suited for infections involving deep renal tissue or calculi [9]. Our patient's clinical improvement after switching from cefdinir to levofloxacin aligns with this pharmacologic reasoning and reinforces the need to individualize therapy based on both drug properties and patient-specific factors, such as neurogenic bladder or nephrolithiasis. However, definitive attribution of the observed clinical course to cefdinir's pharmacokinetic properties is limited by the absence of complete microbiologic susceptibility data, and clinical improvement may have reflected the combined effects of antimicrobial therapy and concurrent ureteral stent placement. At present, few guidelines address empiric antibiotic selection for patients with quadriplegia and concurrent kidney stones, underscoring a gap in the literature and the need for additional studies to help guide clinical practice. While this case is hypothesis-generating rather than practice-changing, it underscores the need for future research to better inform empiric oral antibiotic selection in high-risk populations.

## Conclusions

Patients with quadriplegia and chronic constipation are more prone to a repeating cycle of urinary stasis, infection, and stone formation that complicates their clinical management. When pyelonephritis occurs in the setting of nephrolithiasis, antibiotic selection should account for an antibiotic's ability to reach infected renal tissue as well as the ability to penetrate biofilms. In our patient, a relapse following utilization of cefdinir, followed by resolution after switching to levofloxacin, emphasizes the value of selecting antibiotics based on pharmacologic properties. For individuals with neurogenic bladder and recurrent stones, pharmacologics with strong renal and tissue penetration, such as fluoroquinolones, may offer superior outcomes when used appropriately. Antibiotic pharmacokinetics should be considered when managing infections in patients with altered urinary dynamics.
